# Exploring the Complexity and Promise of Tumor Immunotherapy in Drug Development

**DOI:** 10.3390/ijms25126444

**Published:** 2024-06-11

**Authors:** Yiyuan Feng, Caiying He, Chang Liu, Bingjie Shao, Dong Wang, Peijie Wu

**Affiliations:** School of Basic Medical Sciences and State Key Laboratory of Southwestern Chinese Medicine Resources, Chengdu University of Traditional Chinese Medicine, Chengdu 611137, China; yoravina@163.com (Y.F.); h957279949@163.com (C.H.); 18207217072@163.com (C.L.); shaobingjie2023@163.com (B.S.)

**Keywords:** tumor therapy, immunotherapy, tumor microenvironment (TME), immunosenescence

## Abstract

Cancer represents a significant threat to human health, and traditional chemotherapy or cytotoxic therapy is no longer the sole or preferred approach for managing malignant tumors. With advanced research into the immunogenicity of tumor cells and the growing elderly population, tumor immunotherapy has emerged as a prominent therapeutic option. Its significance in treating elderly cancer patients is increasingly recognized. In this study, we review the conceptual classifications and benefits of immunotherapy, and discuss recent developments in new drugs and clinical progress in cancer treatment through various immunotherapeutic modalities with different mechanisms. Additionally, we explore the impact of immunosenescence on the effectiveness of cancer immunotherapy and propose innovative and effective strategies to rejuvenate senescent T cells.

## 1. Introduction

Cancer remains a major public health challenge globally, with tens of millions diagnosed annually [1]. In many countries, it ranks as the second leading cause of death following cardiovascular diseases. Moreover, the incidence and mortality of cancer are expected to rise due to population aging and lifestyle changes [2]. Current cancer treatments encompass immunotherapy, biotherapy, adjuvant chemotherapy, surgery, and diverse drug combinations, including concurrent chemotherapy and radiation therapy or sequential neoadjuvant chemotherapy [3].

Research indicates that for certain cancers, such as colorectal cancer(CRC), primary treatments like chemotherapy and surgery cannot completely eradicate tumors [3]. Post-surgery, patients face a continual risk of cancer recurrence, impacting their quality of life [4]. The adverse effects of chemotherapy, including potential liver damage such as steatohepatitis, steatosis, and sinusitis, can lead to severe outcomes like liver failure [5,6]. Moreover, chemotherapy may harm healthy cells. Over the past fifty years, cancer treatment strategies have evolved from using highly toxic agents like nitrogen mustard to more tolerable targeted therapies [7]. Nevertheless, challenges such as chemotherapy resistance and recurrence persist, necessitating the exploration of novel therapeutic strategies [8]. One of the prominent therapeutic approaches today is tumor immunotherapy, which has shown promising therapeutic effects [9]. Compared to chemotherapy, immunotherapy is associated with fewer cytotoxic side effects [10] and offers new hope for tumor treatment by targeting specific proteins expressed by cancer cells.

Immunotherapy seeks to enhance immune activity and modulate the immune system to eliminate cancer cells. This innovative approach is proving beneficial for various cancers. This review will detail the advantages and recent clinical progress of tumor immunotherapy, providing a comprehensive summary of new or repurposed drugs based on immunotherapy in recent years. Additionally, it will address immune senescence, a significant factor in tumor development and treatment, discussing its processes, markers, and targeted therapeutic strategies. This aims to surpass the current limitations of tumor immunotherapy and unlock new therapeutic possibilities for cancer patients.

## 2. Immunotherapy: Booming in Tumor Treatment

The immune system plays a pivotal role in detecting cancer and halting its progression [11]. To fully understand the central role of immunotherapy in cancer treatment, it is essential to comprehend the interactions between the immune system and cancer cells comprehensively. The immune system, a complex network of cells, distinguishes between self and non-self, thus protecting the body against both endogenous and exogenous diseases. It also regulates other bodily systems, including the inflammatory response, coagulation mechanisms, and immune surveillance. The immune system comprises white blood cells, lymphoid tissues, and organs such as the bone marrow, lymphatic vessels, lymph nodes, tonsils, spleen, and thymus, effectively recognizing and eliminating a broad spectrum of threats to maintain homeostasis. These functions extend to regulating fluid balance, eradicating harmful microorganisms, and suppressing or destroying cancer cells [12,13]. Unlike chemotherapy, which destroys cancer cells through cytotoxic activities, immunotherapy activates the host’s immune response, enhancing the immune cells’ ability to identify and eliminate tumor cells. This strategy has not only transformed the treatment of certain cancers but has also emerged as a favored strategy in recent years, clinically verified to be effective against a wide range of malignant tumors [14,15].

### 2.1. Definition and Classification of Immunotherapy

Immunotherapy is a method of enhancing or rebuilding the immune system through the use of specific substances to prevent and combat disease. This therapy targets tumors by introducing drugs or agents that stimulate the immune system to generate an immune response. The objective is to optimize the immune system’s function to eradicate cancer cells and augment immune activity, while avoiding an uncontrolled autoimmune inflammatory response that could compromise treatment effectiveness [16].

Immunotherapy includes a diverse array of therapies based on antibodies and T-cell transfer, such as monoclonal antibodies, oncolytic viral therapies, cancer vaccines, cytokine drugs, checkpoint inhibitors, and chimeric antigen receptor (CAR) T-cell therapies, classified by their mechanism of action. Generally, immunotherapy is divided into two types: passive and active. Active immunotherapies, like oncolytic vaccines, are capable of inducing direct, lasting immune-memory responses. In contrast, passive immunotherapies, such as monoclonal antibodies, elicit a targeted response that is typically transient, necessitating frequent administration [17]. Response to immunotherapy, at both radiologic and clinical levels, can be variable, often due to the extended time needed for T cells to mount an immune response and eliminate the tumor. The enduring immune-memory characteristic of immunotherapy offers several benefits, including prolonged immune effects post-treatment, potentially leading to continued anti-tumor activity and enhanced long-term survival. Despite chemotherapy’s effectiveness in killing cancer cells, residual immune deficiencies may increase the risk of recurrence. Immunotherapy addresses this risk by boosting resistance [17].

### 2.2. Dynamics of T Cells in Immunotherapy

T cells, as fundamental components of the immune system, are crucial for the effective recognition and elimination of tumor cells through immunotherapy. A reduction in T cells not only impedes the primary function of immune checkpoint inhibitors but also impacts the resistance pathways of cellular immunotherapy, thereby diminishing the clinical efficacy of immunotherapy and patient quality of life [18,19]. Thus, comprehending the dynamics of T cells during cancer progression is essential for advancing immunotherapeutic strategies. T-cell depletion is a differentiation process triggered by chronic antigen exposure, leading to the continuous stimulation of the T-cell receptor (TCR) and downstream Ca^2+^ signaling, which activates the transcription factor nuclear factor of activated T-cells (NFAT). This activation of NFAT enhances the expression of various inhibitory immune checkpoint proteins (including PD-1, CTLA4, and CD39), resulting in a progressive decrease in T cell effector function and a diminished anti-tumor capacity of the body [20]. The spectrum of T-cell depletion ranges from highly proliferative T-cells with stem cell-like properties (referred to as “precursor-depleted T-cells”) to T-cells that have entirely lost their effector functions and replicative abilities (often termed “terminally depleted T-cells”). The depletion of T cells leads to a reduced expression of co-stimulatory markers and diminished production of inflammatory cytokines (e.g., IFNγ and TNF), further weakening the body’s anti-tumor capacity [21,22]. Overall, the extent of T-cell exhaustion is gauged by the expression of PD-1 on intratumoral lymphocytes, which is strongly associated with cancer patient survival. Exploring factors that influence T-cell status opens new avenues for the enhancement of cancer immunotherapy [23,24,25,26,27].

### 2.3. Advantages of Immunotherapy

In oncology, the advancement of immunotherapy and precision medicine is flourishing. Over the past 15 years, the emergence and ascent of cancer immunotherapy have not only transformed clinical oncology practices but have also significantly enhanced our understanding of cancer’s biological mechanisms [28]. Today, immunotherapeutic agents are indispensable in cancer treatment, offering new options for an expanding patient population. Cancer affects a vast number of people globally, and sustained, detailed research in oncology has greatly advanced our knowledge of cancer and the immune system. A range of clinically validated immunotherapies has altered traditional treatment paradigms and revolutionized tumor immunology. Effective treatments, such as adoptive T-cell therapy (ACT) and immune checkpoints inhibitors (ICIs), are now routinely used in clinics, providing new hope, particularly for patients unresponsive to chemotherapy. These therapies are acknowledged as vital in extending the lives of many patients [29,30,31]. Intensive ongoing research aims to further improve the efficacy of immunotherapy in cancer management. Advances in technology have deepened our understanding of the molecular dynamics of various malignancies and their interaction with the human immune system, leading to innovative therapeutic approaches. Some of these approaches have converted previously lethal cancers into manageable or even curable conditions. Recent immunotherapy research has concentrated on identifying tumor antigens to bolster clinical anti-tumor immunity. Several immunotherapies have been officially sanctioned for cancer treatment.

## 3. Immunotherapy via Diverse Mechanisms

### 3.1. ICIs Based on Various Targets 

Effective anti-tumor responses necessitate moderate T cell activation, which depends on two crucial signals: the antigen-specific signals mediated by the TCR and costimulatory signals from a combination of stimulatory and inhibitory receptors [32]. Immune checkpoints (ICs), key regulators in T cell activation and tolerance, modulate the immune response through co-inhibitory or co-stimulatory signals [33], helping to limit host damage, and are vital for maintaining immune homeostasis and self-tolerance. However, prolonged exposure to neoantigens and continuous T-cell stimulation can lead to an increased expression of immune checkpoints, eventually causing T-cell exhaustion. This mechanism, mediated by immune checkpoints, promotes tumor immune escape. Currently, immunotherapeutic strategies targeting these checkpoints focus on blocking co-suppressive T cell signaling to reinvigorate effective anti-tumor immune responses. The inhibitors targeting these checkpoints are monoclonal antibodies designed to treat tumors and are referred to as immune checkpoint inhibitors [34]. Over twenty monoclonal antibodies targeting PD-1, PD-L1, CTLA-4, and LAG-3 are now approved for treating various types of cancer, including the latest market entries and some repurposed older drugs (Table 1).

#### 3.1.1. Targets PD-1 and PD-L1

**Figure 1:**
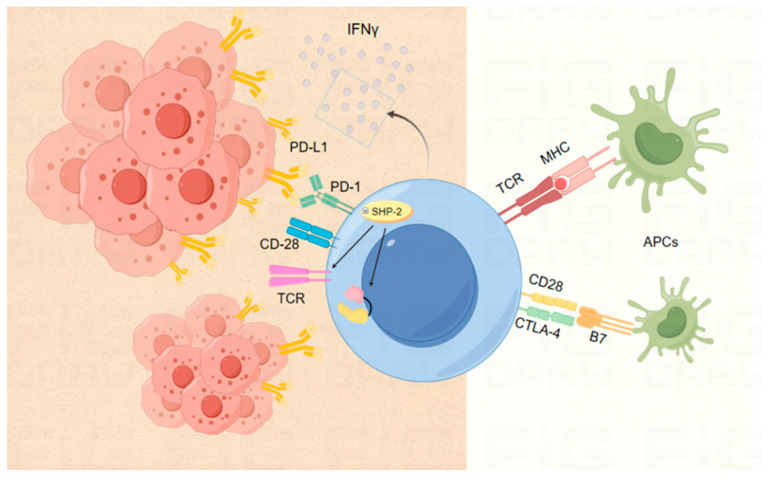
Diagram of the mechanism of action of the ICs PD-1/PD-L1 and CTAL-4. In the tumor microenvironment, TCRs on the surface of T cells bind to homologous peptide-MHC complexes on target tumor cells. However, activation of PD-1 is inhibited when it interacts with PD-L1 on the surface of tumor cells. PD-1 transmits inhibitory signals directly to T cells via SHP-2 phosphatase activation, which suppresses signaling downstream of the TCR and CD28. IFNγ, produced by activated T cells, enhances PD-L1 expression on target cells, creating a negative feedback regulatory mechanism. T cell activation relies on the interaction between B7 ligands on antigen-presenting cells and CD28 receptors on T cells to generate costimulatory signals. CTLA-4, expressed on T cells, moves to the cell membrane and competes with CD28 for ligand binding, thus inhibiting the antigen-presenting cell’s ability to further activate T cells. Abbreviation description: CTLA-4: cytotoxic T lymphocyte antigen 4; IFNγ: interferon gamma; PD-1: programmed death 1; PD-L1: programmed death ligand 1.

When T cells involved in the tumor response are activated, PD-1 expression is upregulated. This upregulated PD-1 binds to its ligands, PD-L1 or PD-L2, which are abundantly expressed on the surfaces of tumor cells, thereby impairing antigen presentation to T cells and interrupting the antigen presentation phase of the tumor immune process (Figure 1). The interaction of PD-1 with its ligands, PD-L1 and PD-L2, on tumor-infiltrating lymphocytes (TILs) is widely regarded as a critical mechanism for tumor immune evasion, making it a crucial therapeutic target. Unlike typical ligand-receptor interactions, which are unidirectional, PD-L1, the primary ligand for PD-1, can also receive inhibitory signals from PD-1, demonstrating a complex regulatory mechanism in T cell signaling along the PD-1 axis [35,36].

Additionally, a range of drugs targeting PD-1 and PD-L1, including Pembrolizumab, Cemiplimab, Dostarlimab, Retifanlimab, Durvalumab, and Atezolizumab, have been approved for treating recurrent head and neck squamous cell carcinoma, CRC, NMIBC, endometrial cancer, and various other solid tumors.

#### 3.1.2. Target CTLA-4

During T cell activation and proliferation, CTLA-4 and CD28 of the B7 family of receptors assume negative and positive roles, respectively, in the initial phases of the immune response [37]. Within the lymph node, CTLA-4 on the surface of T cells supplants the stimulatory receptor CD28 and binds to the ligands CD80 and CD86 [38,39,40,41], a process that leads to a reduced reactivity of antigen-presenting cells (typically denatured cells), thereby preventing further T cell activation [39,41] (Figure 1). Consequently, by blocking CTLA-4 at an early stage of the T cell maturation process, we aim to enhance the anti-tumor immune response [42].

#### 3.1.3. Co-stimulation or Co-inhibition of Other Immune Checkpoints

In addition to well-known ICs like PD-1 and CTLA-4, numerous preclinical and clinical studies are targeting T-cell co-suppressor or co-activator molecules. Most of these molecules display detrimental immunomodulatory effects in cancer contexts, while those with beneficial immunomodulatory impacts are being explored for cancer immunotherapy applications [43].

Interactions between lymphocyte activation gene-3 (LAG-3 or CD223), T-cell immunoglobulin, and mucin domain-containing-3 (TIM-3), and their various ligands (e.g., Galectin-9, HMGB1, PS, and CD66a) diminish anti-tumor immunity through mechanisms such as inducing CD8^+^ T cell death and exhaustion [44,45]. Research has shown that LAG-3 not only inhibits CD8^+^ T cell function but also bolsters the immunosuppressive capabilities of regulatory T cells (Tregs) [46]. In March 2022, the FDA approved Relatlimab, a human lgG4-type LAG-3 monoclonal antibody developed by Bristol-Myers Squibb, for treating unresectable or metastatic melanoma in combination with the anti-PD-1 antibody Nivolumab [47]. This approval highlights LAG-3’s potential as a promising tumor immunotherapy target, succeeding PD-1 and CTLA-4. Other ICs like T-cell immunoglobulin and ITIM domain (TIGIT), V-domain Ig suppressor of T cell activation (VISTA), interacting with ligands such as CD155 and CD112, VSIG-3, exhibit immunosuppressive effects, and inhibitors targeting these have shown significant clinical promise [48,49]. Additional inhibitory checkpoint molecules under investigation include sialic acid-binding Ig-like lectin 15 (Siglec-15) (NCT03665285), B and T lymphocyte attenuator (BTLA or CD272) (NCT04137900), and B7-H3 (CD276) (NCT02628535, NCT03406949).

Conversely, co-stimulatory molecules like inducible T cell co-stimulator (ICOS), glucocorticoid-induced TNFR-related protein (GITR), and OX40, expressed on various immune (e.g., T cells, Tregs, and NK cells) and non-immune cells (e.g., endothelial cells), are being evaluated for their potential in cancer immunotherapy. These molecules not only enhance the functionality of immune cells such as CD8^+^ T cells and Tregs but also regulate helper T cell responses by reducing T cell apoptosis [50,51]. Currently, trials involving ICOS agonist monoclonal antibodies, both as standalone therapies and in combination with anti-PD-1 or anti-CTLA-4 therapies, are underway.

#### 3.1.4. Double Immunotherapy

Despite unprecedented advances in cancer treatment with ICI, the proportion of patients deriving clinical benefits remains limited [43]. To address the shortcomings of individual ICI therapies, researchers are utilizing established and emerging diagnostic strategies, such as genetic and immune biomarkers, to more precisely predict treatment efficacy and to counteract drug resistance through combination therapies.

Compared to monotherapy, combination therapy presents several advantages: it acts on multiple pathways simultaneously, induces synergistic effects, circumvents dosage constraints, and mitigates drug resistance. 

### 3.2. Adoptive Cell Therapy (ACT)

ACT involves harvesting immune cells from a patient’s body, culturing and expanding them in vitro, enhancing their targeted killing function, and then reinfusing these modified cells back into the patient. This process aims to eliminate pathogens, cancerous cells, and mutated cells from the blood and tissues. Currently, therapies that enhance natural anti-tumor activity (such as immune checkpoint blockade) and those that administer specific anti-tumor immune cells (overt T-cell therapy ACT) have received extensive clinical validation and approval. Although both approaches aim to stimulate specific anti-tumor responses in T cells, the efficacy of ICIs is limited by the patient’s own immune tumor-reactive T cell population and may be less effective against less immunogenic cancers. In contrast, ACT, by administering tumor-specific T cells, can recognize these poorly immunogenic cancer types and has the potential to enhance the therapeutic efficacy of ICIs [52]. Among ACT therapies, the more researched therapies are CAR-T, CAR-NK, TCR-T, and TILs therapies, which differ in terms of cell source, principle of action, durability and infiltration capacity (Table 2). Over a dozen ACT drugs have been approved for marketing in China, and recent significant breakthroughs have been made in the U.S. in drug studies on TILs, TCR-T, and CAR-T (Table 3).

#### 3.2.1. TILs

**Figure 2:**
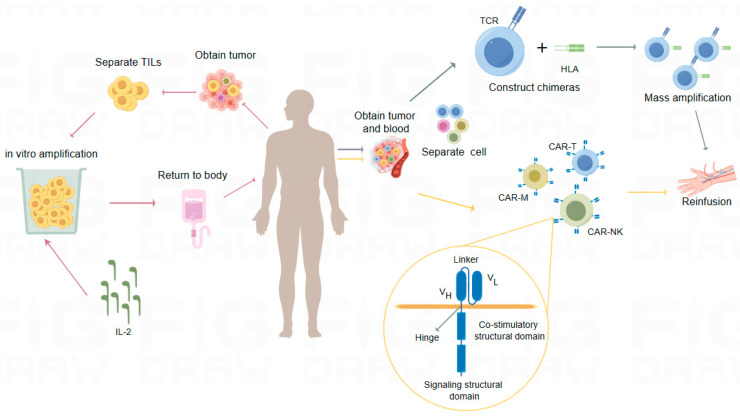
The three modalities of the sequential therapies. TIL therapy: TILs are extracted from tumor samples, expanded in vitro, and infused into lymphocytopenic patients. In this process, IL-2 serves as a T-cell growth factor, enhancing the in vitro expansion and efficacy of TILs. TCR-T therapy: TCR-T involves the transduction of chimeric antigen receptors (combining antigen-binding domains and T-cell signaling structural domains) or TCRα/β heterodimers into normal T cells. These TCR-Ts are then expanded in large quantities ex vivo and reinfused into the patients. CAR-based therapies: An artificially designed CAR molecule is introduced into T cells, NK cells, M cells, etc., to confer targeted functionality. These modified CAR-T cells are then infused back into the patient for treatment. The CAR molecule comprises three main parts: the extracellular domain, the transmembrane domain, and the intracellular domain. The antigen-binding domain within the extracellular domain typically consists of an antibody-derived single-chain fragment (scFv), which includes the variable light (VL) and heavy (VH) chains of the antibody connected by a linker, and attaches to the transmembrane structural domain through a hinge. The intracellular domains include a co-stimulatory structural domain and a signaling structural domain, essential for the complete activation of T cells. Expression abbreviations: TILs: tumor-infiltrating T cells, TCR: T cell antigen receptor, CAR: chimeric antigen receptor T cell immunotherapy, IL-2: interleukin-2, VL: variable light chain, VH: variable heavy chain, Hinge: hinge region.

TILs are isolated from surgically resected tumor samples and expanded in vitro before being infused into lymphocytopenic patients (Figure 2). The use of interleukin-2 (IL-2), a T-cell growth factor, has enabled large-scale in vitro expansion of TILs and demonstrated increased lethality against tumor cells in conditions such as melanoma, HCC, lung carcinoma, ovarian cancer, and other solid tumors [53]. In February 2024, the FDA approved a global TIL cell product called lifileucel for the treatment of adult patients with unresectable or metastatic melanoma who have previously received PD-1 antibody therapy. This approval marks the first commercially available TIL therapeutic and represents a significant advancement in the treatment of solid tumors. If the tumor is BRAFV600-positive, treatment may include a BRAF inhibitor, with or without an MEK inhibitor. This approval was based on the Phase II C-144-01 study, where lifileucel showed clinically meaningful activity in heavily pretreated patients with advanced melanoma and a high tumor burden [54]. Iovance Biotherapeutics sponsored a clinical trial that led to lifileucel’s approval. The FDA approved lifileucel dose was administered to over 70 participants, and nearly one-third showed at least some reduction in size, with many tumors disappearing completely. A long-term follow-up study indicated that lifileucel achieved an ORR of 31.4%, compared to 31.3% for anti-PD-1/PD-L1 therapy in primary refractory patients based on the study criteria [54]. 

#### 3.2.2. T-Cell Receptor Chimeric T-Cell Therapy (TCR-T)

Also known as TCR engineered T-cell therapy, TCR-T involves screening for suitable tumor-specific antigens and highly specific TCRαβ chain sequences. These target TCR sequences are then introduced into T cells, where the imported TCRα and β chains dimerize and complex with endogenous CD3 components [55,56]. Typically, these sequences are cloned into retroviral or lentiviral vectors for the in vitro transduction of patient peripheral blood T cells, producing TCR-T cells that specifically recognize tumor antigens [57] (Figure 2). These cells are then expanded and re-infused into the patient’s body to specifically eliminate tumor cells.

In 2022, the first TCR-T cell therapy product, “Tebentafusp (tebentafusp-tebn/Kimmtrak)”, was approved by the FDA for the treatment of uveal melanoma. This marked the first bispecific T-cell engager (TCE) to be successfully approved. The Phase III clinical study of Tebentafusp enrolled 378 patients with metastatic uveal melanoma and demonstrated that the Tebentafusp group achieved a significant improvement in OS compared to other treatment groups (including Dacarbazine, Ipilimumab, or Pembrolizumab), with a one-year survival rate of 73 percent in the Tebentafusp group versus 58 percent in the comparison groups [58]. While monotherapy with TCEs has shown significant results in the treatment of hematologic cancers, it has underperformed in the treatment of solid tumors. However, the approval of Tebentafusp confirms the efficacy of TCEs in combating solid tumors, which is of great significance [54].

#### 3.2.3. Cell Therapy Based on CAR Technology

CAR technology features a switch molecule composed of a specific antigen-binding scFv and a paired cognate leucine zipper. This arrangement includes a universal receptor linked to intracellular signaling domains (Figure 2). In the field of CAR therapies, the development extends beyond T cells to include other immune cells such as CAR-NK, CAR-macrophage (CAR-M), CAR-γδT, and CAR-NKT, which have also garnered extensive attention.

As the pioneering CAR therapy, CAR-T merges the specific binding capabilities of the extracellular single-chain variable region of immunoglobulins with the activation potential of the intracellular region of the TCR to create an artificial chimeric antigen receptor (CAR). This CAR is then transduced into autologous cytotoxic T-lymphocytes to form CAR-T cells [59,60]. Unlike TCRs, which recognize only HLA-restricted antigens and depend on co-stimulatory factors such as CD3, CD4, and CD8 for T-cell activation, CAR-T can identify both cell surface and extended antigens. Since 2017, the FDA has approved five CAR-T products for treating hematologic tumors. However, the use of CAR-T in solid cancer treatment remains limited due to the TME and immunosuppression, with only two CAR-T therapies, Yescarta and Kymriah, approved for lymphoma treatment [61]. Notably, CAR-T treatments are associated with severe side effects, including cytokine release syndrome and neurotoxicity. To overcome these issues, researchers have developed various strategies, including optimizing CAR constructs and exploring innovative therapeutic combinations to enhance the specificity, infiltration, and efficacy of CAR-T cell therapy [62].

From 2023 to early March 2024, three CAR-T therapy products were launched in China: fully human targeted BCMA CAR-T Igibiorense injection, CNCT19 cell injection, and Zevor-cel. These products are approved for treating relapsed or refractory multiple myeloma (r/r MM), adult relapsed or refractory B-cell acute lymphoblastic leukemia (r/r B-ALL), and relapsed or refractory multiple myeloma. Before this, six CAR-T therapies had received FDA approval, including Novartis’ Kymriah (for ALL and DLBCL), Gilead/Kite’s Yescarta (for DLBCL) and Tecartus (for MCL), and Bristol-Myers Squibb’s Breyanzi (for DLBCL) and Abecma (for r/r MM).

CAR-NK therapy is distinguished by its strong anti-tumor activity, high safety profile, diverse sources, and multiple targets [63]. Its cytotoxicity is comparable to that of CD8-positive cells, with the ability to release both perforin and granzyme. Compared to T-cell therapy, NK cells present a lower risk of inducing graft-versus-host disease (GVHD), making CAR-NK therapy more tolerable for patients undergoing adjuvant therapies such as cytotoxic chemotherapy, radiotherapy, and molecularly targeted therapies [64]. CAR-M therapy offers several advantages including high intra-tumor migratory capacity, both antigen-dependent and -independent phagocytosis, enhanced antigen presentation, and the remodeling of the immunosuppressive microenvironment. Macrophages contribute to tumor cell elimination through phagocytosis, and the release of reactive oxygen species and nitrogen (ROS/iNOS), while degrading almost all components of the ECM [65,66]. The unique phagocytosis properties of CAR-M therapies may offer greater benefits in the treatment of solid tumors compared to CAR-T and CAR-NK cell therapies. CAR-γδT and CAR-NKT cell therapies exert their effects through various mechanisms, including CAR-mediated killing, antibody-dependent cellular cytotoxicity (ADCC), Fas/FasL, TRAIL, and TNFR pathways. These therapies kill tumor cells and secrete cytokines to activate other immune cells, demonstrating strong anti-tumor activity with minimal immunosuppressive effects [67]. In recent years, a number of ongoing clinical trials of ACT therapies (Table 4) have highlighted the significant potential and promising future of immune cell therapy as an effective treatment option for patients.

### 3.3. Cytokine Therapy

Cytokines play a crucial role as mediators in the TME, with cytokines involved in adaptive immune responses (e.g., IFNγ, IL-2, IL-12, and TNF) positively impacting anti-tumor immunity.

IL-2 stimulates T-cell responses, natural killer cell proliferation, CD4^+^ T-cell activation, and B-cell antibody production. It is currently approved by the FDA for treating patients with RCC and melanoma in stable stages [68]. High doses of IL-2 have induced anti-tumor immune responses in select cancer patients. Moreover, when combined with the immune checkpoint blocker nivolumab, IL-2 significantly inhibits tumor growth in cancers such as melanoma, RCC, NSCLC, uroepithelial carcinoma, and triple-negative breast cancer (TNBC) [69].

IL-12 promotes NK and T cell proliferation and cytotoxicity, enhances T1 cell differentiation, and stimulates IFNγ production [70]. Challenges related to its transient half-life and toxicity can be mitigated through the targeted or localized delivery of IL-12 [71].

IFNγ induces anti-tumor responses through CD4^+^ T helper cells and CD8^+^ T cells. Research has shown that IFNγ enhances immunosuppressive molecule regulation, tumor MHC expression, and T cell infiltration. Currently, combination therapies involving recombinant IFNγ and immune checkpoint inhibitors like pembrolizumab and nivolumab are under development [72].

Tumor necrosis factor (TNF) promotes tumor activity through various pathways, including cytokine cascade stimulation, fibrotic response induction, and the alteration of adhesion receptors. Studies have shown significant effects of antibody therapies targeting TNF or using soluble TNF receptor fusion protein traps in treating solid tumors [73,74]. Additionally, therapeutic strategies targeting cytokines such as IL-7, IL-15, IL-10, IL-6, and TGF-β have demonstrated promising results in solid tumor clinical trials.

### 3.4. Cancer Vaccines

Cancer vaccines elicit a strong anti-tumor cytotoxic T-cell response by exposing the immune system to tumor-associated antigens (TAA) or tumor-specific antigens (TSA), which recognize and destroy cancer cells. The first and second HPV vaccines were approved by the FDA in 2006 and 2010, respectively. In 2010, Sipuleucel-T (Provenge, Previx), an autologous dendritic cell (DC)-based prostate cancer vaccine, was recognized by the FDA as the first approved therapeutic in tumor vaccines [74,75]. Depending on their preparation, cancer vaccines can be categorized as cellular, peptide, viral, or gene vaccines.

There are two types of cellular vaccines: autologous or genetically modified tumor cells and activated DC vaccines. Tumor cell vaccines contain a complete complement of TAA, including epitopes for CD4^+^ T cells and CTLs. DCs activate naïve T cells to stimulate an immune response and collaborate with other immune cells. The development of anti-TME vaccines involves preparing DCs in large quantities and loading them with various TAAs or adjuvants to enhance the immune response against tumors [76]. 

Viral vaccines are the most effective method for inducing cellular and, to a lesser extent, humoral immune responses [77]. In 2015, the FDA authorized T-VEC for the treatment of melanoma, marking it as the first FDA-approved oncolytic viral-based therapy. This therapy regulates both local and systemic immune responses against tumors through the direct lysis of cancer cells and “vaccine-in-place” effects. 

Peptide vaccines are designed to identify peptides in pathogens that trigger an immune response, integral to producing the vaccine material. One type of anticancer vaccine derived from peptides is the synthetic long peptide (SLP), which induces new antigen-reactive CD8^+^ and CD4^+^ T cell responses [78]. In contrast, recombinant overlapping peptides can break self-tolerance and generate superior immunogenicity in CD4^+^ and CD8^+^ T cells compared to native proteins.

Genetic vaccines are categorized into DNA vaccines and mRNA vaccines. DNA vaccines are heat stable, easy to store and transport, and facilitate mass production, ensuring affordability [79]. They can induce both humoral and cellular immunity, are easy to manufacture, and can provoke prolonged immune responses, especially those involving antigen-specific CTLs. The enhancement of DNA vaccine immunogenicity can be achieved through several strategies: improved delivery mechanisms, enhanced DNA stability in vivo, and the concurrent delivery of DNA encoding cytokines. Compared to DNA vaccines, mRNA vaccines, which are translated in the cytoplasm and require only membrane traversal, offer a higher level of safety [80]. Cancer vaccines such as the hepatitis B vaccine, human papillomavirus vaccine, and prostate cancer vaccine have already been approved and are widely used in clinical settings.

## 4. A Key Player in Influencing the Development and Treatment of Cancer: Immunosenescence

Although cancer immunotherapies are now considered the cornerstone of cancer treatment, there are notable limitations. First, only a small fraction of patients benefit from these therapies, largely due to the significant constraints posed by immunotherapy-related adverse events (irAEs) [43]. Moreover, the types of irAEs occurring after checkpoint blockade do not appear to be cancer-specific, suggesting that the etiology of irAEs is a drug-induced loss of immune tolerance unrelated to the tumor itself [81,82]. Second, some tumors initially responsive to immunotherapy may eventually develop resistance. Numerous studies are currently evaluating the effectiveness of treatments to counteract this resistance. Additionally, the high cost of immunotherapy, even in developed countries, poses a substantial economic burden, challenging for both society and most patients [83]. Furthermore, the effects of immunotherapy are not long-lasting; only about one-third of patients experience enduring benefits, a predicament exacerbated by the diversity of immune types and more daunting than acquired treatment resistance. Even when combined with other therapies, long-lasting effects are achieved in fewer than half of the patients. This limitation underscores the vast challenges facing oncology treatment and highlights the critical need for ongoing exploration and innovation in therapeutic strategies. Clinical studies have indicated that older cancer patients, particularly those over 75, typically experience poorer clinical outcomes compared to younger patients, primarily due to age-related comorbidities, declining immune function, and reduced tolerance to treatment side effects. Age-related immune senescence, especially the decline in T-cell function, may result in lower levels of tumor-infiltrating immune cells (TIICs), which in turn curtails the efficacy of cancer immunotherapy to some extent. To address this bottleneck in tumor immunotherapy, we explore the process of immune senescence and its associated markers, examine how immune senescence impacts cancer progression and tumor immunotherapy, and propose new strategies to rejuvenate senescent T cells and modulate their upstream pathways.

With increasing human life expectancy and a trend toward population ageing, the risk of age-related diseases, including tumors, is rising in the elderly population. Concurrently, there has been an increase in the failure rate of immunotherapy and the rate of relapse after treatment [84,85]. This phenomenon, known as immunosenescence, was first proposed by Roy Walford [86,87,88,89,90]. Research has shown significant differences in anti-tumor immune responses between younger and older patients. For example, Palmer et al. conducted extensive research on the age distribution of 100 different tumors and concluded that the immune system plays a crucial role in tumor development [91]. Additionally, Vatter et al. performed a phenotypic analysis of breast epithelial cells in 57 women aged 16 to 91 years and found that the accumulation of luminal and progenitor cells may increase the risk of cancerous transformation with age [91]. The incidence and prevalence of most cancers increase with age as tumor escape mechanisms intensify and immune surveillance diminishes. This rising risk is due to a combination of factors that together influence an individual’s susceptibility to cancer. Among these factors, the continuous accumulation of senescent cells in tissues, coupled with reduced immune cell function and proliferative potential, are key components of immune senescence.

### 4.1. The Process of Immunosenescence and Related Markers

**Figure 3:**
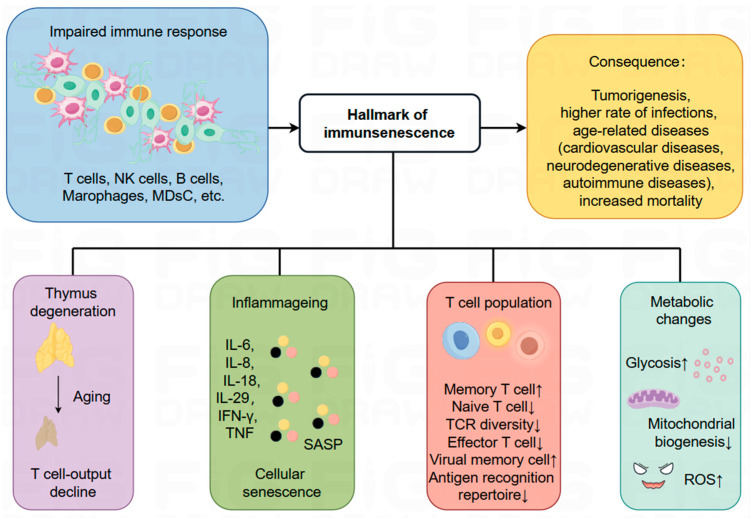
The process of immune senescence and associated markers. The aging of the immune system can alter immune responses, potentially leading to the development of various diseases such as tumors, infections, cardiovascular diseases, neurodegenerative diseases, autoimmune diseases, and metabolic disorders. Concurrently, significant changes occur within various immune cell subpopulations, particularly in T cell subsets. These changes include reduced T cell production due to thymic degeneration, abnormal T cell metabolism, and altered ratios of T cell subpopulations. As the immune system ages, metabolic alterations also occur, such as a SASP-mediated chronic low-grade inflammatory environment, impaired response to neoantigens, increased glycolysis, mitochondrial dysfunction, and elevated production of ROS (In the Figure 3 “↑” represents “increase” and “↓” represents “decrease”).

Immunosenescence is an aging process characterized by immune dysfunction, involving the remodeling of multiple organs and cellular-level changes that significantly impact the immune system. These changes lead to progressive alterations in the innate and adaptive immune systems, increasing the risk of diseases such as tumors and infections, and may result in a diminished response to infections or vaccines in older adults [92]. Immunosenescence is a dynamic and multifactorial biological process influenced by factors including aging itself, chronic inflammation, and microenvironmental changes (Figure 3).

As the immune system ages, it undergoes adaptive metabolic changes. While the biological specifics of these changes are not fully understood, several characteristic alterations have been observed. These include thymic degeneration, the dysfunction of hematopoietic stem cells (HSCs), imbalance in T- and B-cell naïve/memory ratios, increased inflammation, the accumulation of senescent cells, impaired response to neoantigens, heightened glycolysis, mitochondrial dysfunction, and a rise in the production of ROS, genomic instability, and stress responses [93,94]. These modifications are critical for understanding the mechanisms and impacts of immunosenescence, particularly in the context of age-related diseases. Features of immune senescence are closely linked with increased morbidity and mortality from age-related conditions such as metabolic diseases, neurodegenerative disorders, cardiovascular diseases, autoimmune diseases, and cancers in elderly patients [94]. To fully grasp immune senescence and associated diseases, it is essential to explore molecular mechanisms, immune cell dynamics, and regulatory signaling. A deeper comprehension of these biological processes is expected to yield novel insights and strategies for the prevention and treatment of these age-related diseases (Figure 3).

During immune senescence, all types of immune cell subsets, especially T cells, undergo substantial changes. These changes are marked by decreased T cell production, abnormal T cell metabolism, and imbalances in T subset ratios due to thymic degeneration [91,95]. Thymic degeneration is critical in shaping the balance of immune cells, particularly T cells [96,97,98]. The thymus comprises two distinct tissue types: epithelial tissue devoid of thymopoiesis and non-epithelial perivascular space. With the aging of the thymus, the interstitial epithelial layer increasingly disappears, and the perivascular layer progressively fills the thymus. This transition results in a decrease in naïve T cells, an increase in peripheral memory T cells, and a reduced likelihood of newly generated T cells migrating to peripheral tissues [99,100,101].

An important hallmark of immunosenescence is the chronic, low-level “inflammation” throughout the body, characterized by elevated blood inflammatory markers, which is considered a central factor in the aging process [92,102]. This inflammation stems from the accumulation of damaged macromolecules, while chronic tissue damage primarily arises from endogenous cellular debris [103]. Cellular senescence is at the heart of this inflammatory process. According to Effros RB et al., senescent CD8^+^ T cells accumulate in vivo during immune senescence [104]. In these senescent cells, the senescence-associated secretory phenotype (SASP) is manifested through the secretion of numerous soluble factors, including interleukin-1 (IL-1), IL-13, IL-18, IL-6, IL-8, and receptors for TNF. These SASP factors operate in an autocrine manner, creating positive and negative feedback loops and regulating the activity of neighboring cells in a paracrine manner, thereby leading to an inflammatory phenotype and exerting a significant influence on the immune aging process [105,106,107]. Crucially, SASP can propagate senescence, as it contains prohormonal factors released in extracellular vesicles [108], which in turn further propagate SASP [109]. In the context of cancer, SASP significantly affects tumor progression, potentially promoting or impeding it, depending on the specific composition of the SASP [110]. Thus, SASP may act as a double-edged sword in cancer therapy, as immune cells rely on it to mediate anti-tumor responses [111,112,113], promote “senescence surveillance”, and prevent tumorigenesis [114,115]. However, under chronic conditions and pathological states such as established tumors, SASP components like vascular endothelial growth factor (VEGF), CCL5, and IL-6 may induce adverse effects related to cancer, including drug resistance, cancer progression, and cachexia [116,117,118,119,120,121]. Therefore, a deeper understanding of the complex interactions between cellular senescence, pro-inflammatory factors, and aging is critical in deciphering the mechanisms of immune senescence and potentially designing interventions to mitigate the detrimental effects of inflammation and promote healthier aging.

### 4.2. Immunosenescence and Cancer

Immunosenescence is a critical hallmark of aging, strongly associated with cancer and cellular senescence, often leading to a poor prognosis for patients [122]. During senescence, the immune system fails to effectively eliminate emerging senescent cells, resulting in their accumulation. This accumulation promotes the SASP and a pro-inflammatory state in tissues, potentially inducing or accelerating tumor pathology, especially in the elderly population [110]. The elderly are particularly vulnerable to various degenerative and oncological disorders due to the decline in normal cellular functions [92].

T cells are central to the body’s anti-tumor immunity. Research has highlighted that the immune senescence of CD8^+^ T cells is a crucial factor in tumorigenesis and development [123]. Thymic degeneration reduces the number and proportion of naïve CD8^+^ T cells as we age, impairing immune system functionality [124]. This not only increases susceptibility to age-related diseases but also heightens cancer risk in the elderly. Age-related changes in immune responses, including the downregulation of interferon (IFN) signaling in CD8^+^ T cells, have been documented in aged mouse models [125]. In older individuals, immune system imbalances may promote tumor proliferation and accelerate T cell senescence. Concurrently, there is a significant increase in the number of macrophages associated with tumors alongside Tregs [126,127,128,129]. Tumor cells activate both the PKA-CREB and P38 signaling pathways via endogenous cyclic AMP production, leading to DNA damage and T cell exhaustion [130,131]. Senescent T cells enhance immune checkpoint receptor expression and increase PD-L1 expression in tumor cells [132,133]. Glucose exposure activates the ATM and AMPK pathways, further accelerating T cell senescence [134]. Senescent T cells increasingly rely on anaerobic glycolysis for energy, resulting in impaired mitochondrial function and increased ROS production. Additionally, signaling pathways such as cGAS-STING, C/EBPβ, and NFκB are closely linked to T cell senescence [94]. These findings illustrate a complex interplay between aging, immune senescence, and tumor development, shedding light on the intricate effects of age on cancer progression and prognosis.

**Figure 4:**
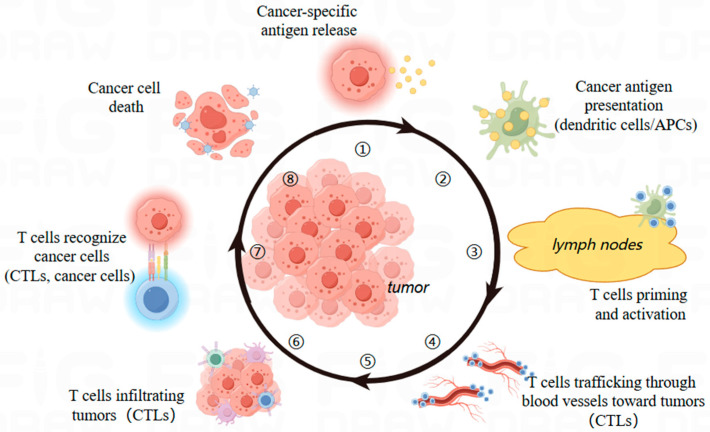
The cancer immune cycle begins with the release of antigens from cancer cells and ends with their destruction, encompassing seven major steps. This theoretical framework was first proposed in 2013 and continues to be a critical component in the study of cancer immunology. The diagram identifies the primary cell types involved and their anatomical locations. Abbreviations: APCs, antigen presenting cells; CTLs, cytotoxic T lymphocytes.

As medical science advances, immunotherapy has emerged as a promising tool to rejuvenate the aging immune system in elderly cancer patients. However, implementing this strategy presents a key challenge: the TME, a complex network that directly impacts the efficacy of immune checkpoint blockade therapy and other novel immunotherapies. As cancer progresses, the TME evolves to suppress immune responses, inhibiting immune cells from destroying malignant or tumor cells. Thus, understanding the immune regulation within the TME is crucial. In the TME, cancer-specific antigens produced during tumorigenesis are processed and captured by DCs. These activated DCs migrate to lymph nodes draining the tumor, where they activate and differentiate naive T cells into effector T cells capable of recognizing and eliminating tumor cells. These activated effector T cells then migrate from the lymph node through the vasculature to the tumor, penetrating the tumor bed in a multi-step process that includes adhesion between vascular endothelial cells and T cells, and migration across the endothelium [135,136]. When T cells infiltrate the tumor bed, their recognition of specific tumor antigens enables them to destroy cancer cells, stimulating further antigen secretion and the successive activation of the cancer immune cycle (Figure 4) [137]. To overcome these challenges, we propose a new strategy—modulating the tumor immune microenvironment. By regulating factors such as cell metabolism, signaling pathways, and immune cell activity within the TME, we aim to enhance the effectiveness of immunotherapy and improve the lifespan and overall quality of life for elderly patients diagnosed with cancer. After extensive clinical trials, several tumor immune microenvironment modulators have demonstrated therapeutic effects. For example, small-molecule PD-1/PD-L1 inhibitors (INCB086550 and IMMH-010), IDO1 inhibitors (LY-3381916 and Indoximod), and TGF-βRI inhibitors (LY-2157299, GW788388, EW-7197, and LY-3200882) can be used alone or in combination with immune checkpoint-targeting drugs to potentially achieve better efficacy [138,139,140,141].

Through a comprehensive analysis and precise modulation of the tumor immune microenvironment, we aim to develop a novel and effective immunotherapy pathway for elderly cancer patients. Recently, the pivotal role of immune senescence in tumor progression has garnered significant attention, necessitating a further exploration of the inherent connection between immune system senescence and the efficacy of immunotherapies. This will aid in optimizing immunotherapeutic strategies for elderly cancer patients.

### 4.3. Impact of Immune Senescence on Tumor Immunotherapy

As the elderly population expands, the significance of immunotherapy for treating older cancer patients increases. Immunotherapy’s effectiveness varies between older and younger patients due to factors such as cancer type, disease stage, and comorbidities. It is critical to thoroughly investigate immunotherapy’s efficacy in this demographic to refine therapeutic strategies and meet the unique challenges posed by aging and cancer. Accordingly, our systematic study specifically examines the efficacy of cancer immunotherapy and its influencing factors in the elderly.

Research indicates that the efficacy of immunotherapy can be predicted by the proportion of senescent T-cells prior to treatment, revealing that immune system aging diminishes the effectiveness of ICI. For instance, in NSCLC, preclinical studies have demonstrated reduced efficacy in aged mouse models compared to younger ones, highlighting substantial age-related variations across different tumor types and physiological conditions [142,143,144]. Furthermore, the effectiveness of ICI in elderly TNBC mice is limited, as observed in specific studies. Additionally, alterations in the TME, such as decreased IFN signaling and antigen presentation, have been noted in these patients [125]. Padron et al. reported that anti-PD-1, anti-CTLA-4, and anti-PD-L1 antibodies were highly effective in young melanoma mouse models [145]. In contrast, older mice showed poor responses to anti-PD-L1 therapy, although anti-PD-1 and anti-CTLA-4 treatments remained effective. Clinical trials have indicated that elderly patients with RCC, uroepithelial carcinoma, and melanoma experience lower progression-free and OS rates compared to younger patients [146]. However, older melanoma models showed more favorable outcomes with anti-PD-L1, anti-PD-1, and anti-CTLA-4 therapies. A retrospective study also found that senior patients (≥75 years old) with advanced melanoma had better therapeutic outcomes with anti-PD1 therapy, suggesting that ICI might offer enhanced efficacy and tolerability in this group [142,147,148,149,150].

The low participation of elderly patients in ICI clinical trials has led to a scarcity of data regarding their safety and toxicity, and the effects of ICI antibody therapy on this demographic remain underexplored. Unraveling the mechanisms that drive rapid tumor progression and affect the effectiveness of immunotherapy in elderly patients is both critical and challenging [151]. Furthermore, to improve the efficacy of cancer immunotherapy and tailor treatments, it is essential to examine the relationship between treatment responses and age, as well as the nuanced differences in the TME. This endeavor is crucial and vital to devising more accurate and effective treatment strategies for patients of all ages [146,152]. Despite numerous challenges and uncertainties in this area, the rapid progress of technology and deepening research provide strong grounds for optimism that these strategies will soon offer new hope to many cancer patients.

### 4.4. Therapeutic Strategies for Immune Aging

In 1939, a pivotal study discovered that caloric restriction in mice and rats effectively extended their lifespan. This was the first evidence that the aging process is modifiable [153]. Subsequent research in primates confirmed that a restricted diet not only prolongs lifespan but also slows the progression of age-related diseases [154,155,156]. Additionally, reducing the number of senescent cells through genetic and pharmacological methods has been shown to prevent and mitigate various age-related disorders [157,158]. Thus, conducting comprehensive research on these interventions and facilitating their widespread implementation in human studies is crucial.

T-cell senescence is a key factor in aging and immune function, significantly affecting immune system efficacy. Simple anti-aging interventions may not yield the desired therapeutic outcomes and could potentially harm healthy tissues, particularly in the elderly. To rejuvenate the T-cell pool, several strategies are viable: first, directly enhancing the degenerating thymus to restore its T-cell production capacity; second, eliminating and replacing senescent T-cells by enabling them to re-enter the cell cycle through cellular reprogramming or extending telomeres; and third, targeting upstream mechanisms that induce senescence [146].

The degeneration of the thymus is a significant aspect of aging, impacting T-cell production and reducing the body’s tumor-fighting capacity. Researchers have explored various methods to rejuvenate the structure and function of the aging thymus. For instance, studies have shown that transplanting cells from young thymic epithelium into aged or defective thymuses enhances thymic regeneration and boosts T-cell production, thereby directly ameliorating thymic degeneration [159]. Similarly, injecting a plasmid vector containing FOXN1-cDNA into the thymus of aged mice has been found to partially restore the thymus size and thymocyte count [160]. This indicates that therapies targeting the FOXN1 gene could potentially revitalize the anatomical structure and physiological function of the aging thymus. Moreover, IL-7 therapy applied to the thymus in older individuals has successfully rejuvenated T-cell development in the elderly [161]. It has been reported that in response to the reduced T-cell production resulting from thymic deterioration, intervention through modulating immunomodulatory factors is possible. Specifically, IL-7 fusion proteins—IL-7 bound to the N-terminal extracellular structural domain of CCR9—have shown the ability to rejuvenate thymic structure in the elderly, indicating significant potential for targeted cytokine therapy (Table 5). Additionally, it has been demonstrated that rejuvenating thymic function in the elderly can be achieved by optimizing the thymic stromal microenvironment.

There is an association between short telomeres, age-related diseases, and reduced lifespan in both mice and humans. Activating telomerase can prevent telomere shortening. In a mouse model, extending telomeres reduced DNA damage and signs of aging, thereby prolonging the lifespan of the mice [164]. Alessio Lanna et al. identified a novel mechanism that extends the lifespan of the immune system: the transfer of telomeres from antigen-presenting cells to receptor T-cells increased the telomeres in the T-cells by an average of about 3000 base pairs, an effect substantially greater than that achieved by telomerase alone [165]. This telomere transfer protects the recipient T-cells from replicative senescence, providing them with enduring immune memory and stem cell characteristics, which enables these T-cells to deliver long-term protection against severe infections. Cyclohexenol has been found to inhibit telomere contraction and boost telomerase activity and proliferation in CD8^+^ T cells. However, strategies for telomere lengthening may carry potential risks in the context of cancer, due to the absence of specific mechanisms targeting senescent cells [166].

By precisely regulating the upstream mechanisms that induce T-cell senescence, we effectively modulated the relevant metabolic pathways, thereby preventing the premature senescence of T-cells and enhancing their immune functions. Treg-mediated senescence regulation strategies show considerable promise. In age-related diseases, the increased proportion of Tregs poses a significant barrier to effective immune responses [167,168]. Studies have shown that Treg-induced T-cell senescence can be effectively inhibited in animal models both in vitro and in vivo by modulating glucose metabolism [169]. At the mechanistic level, poly-G3 activated TLR8 in Treg cells, which subsequently inhibited glucose uptake, transport, and glycolysis. The activation of TLR8 signaling resulted in decreased expression levels of cyclic adenosine monophosphate and mTORC1-HIF-1 signaling, thus reducing metabolic activity and senescence in neoplastic cells. Recent research reveals that senescent T-cell function can be restored via the MAPK pathway. Consequently, by activating TLR8 or inhibiting MAPK signaling in Tregs, we can reverse T-cell senescence both in vitro and in vivo [134]. Furthermore, activating AMPK pathways has emerged as an innovative approach to counteract cellular aging and bolster the aging immune system [126,170]. The continued exploration of novel molecular and cellular mechanisms linked to premature senescence and inflammation will provide a solid theoretical basis for developing new therapeutic strategies. With advanced deep learning tools, we can more efficiently screen anti-aging drugs [171]. Finally, an innovative strategy involves restoring immune function by cryopreserving young, functioning autologous leukocytes and re-infusing these cells later or using them in anti-cancer immunotherapy strategies. This approach offers a novel perspective for combating immune system aging and enhancing its functionality [172].

Recent studies have shown that pharmacologic interventions exhibit some potential in slowing down the aging phenotype. The findings of metformin, a widely used antidiabetic drug, suggest it may play a role in decelerating the aging process [173]. A retrospective analysis of diabetic patients treated with metformin indicated an increase in life expectancy compared to non-diabetic patients [174]. Additionally, beneficial effects on aging have been observed in mouse models. However, these findings were primarily conducted in short-lived mouse models prone to cancer under certain conditions. Subsequent studies observed similar phenomena in longer-lived C57BL/6J mice and genetically inbred mice (Table 5). Alejandro Martin-Montalvo’s recent study demonstrated that adding 0.1% metformin to the diet of male C57BL/6J mice (from week 72 to week 90) increased their average lifespan by 4–6% and improved their health indicators [175,176]. MTOR, a critical protein kinase, plays a role in various signal transduction pathways, including energy status, growth factors, nutrient utilization, and stress response [177]. Numerous studies have shown that the genetic modulation of mTOR signaling can slow the aging process across a variety of organisms [178,179,180]. These signaling regulatory mechanisms, which include mRNA translation, transcription, autophagy, and mitochondrial function, are known to facilitate lifespan extension. Furthermore, the binding of rapamycin to FKBP12 disrupts mTORC1 and inhibits its function, showing promise in slowing or reversing various age-related changes [181,182]. Although rapamycin has been effective in extending lifespan and mitigating major pathological changes in mice through its anti-tumor mechanism and reversing enhanced SASP in senescent cells, studies on the mTORC1 pathway provide the most robust clinical evidence to date, suggesting it as a viable strategy to forestall aging (Table 5) [167,183]. However, it is important to note that the lack of selectivity of rapamycin may lead to serious side effects, including inflammatory responses and potential damage to normal tissues. Thus, a thorough exploration and evaluation of its potential safety issues will be a crucial part of future research and application.

As scientific research advances, we are gaining a more detailed understanding of the underlying molecular mechanisms of aging, which opens new opportunities for anti-aging therapies. The impact of immune senescence on tumor growth and development underscores the need for a further exploration of its specific role in tumor treatment and progression. By modulating the SASP during senescence, we can enhance therapeutic outcomes using combination therapies. For instance, chemotherapy-induced SASP can increase susceptibility to PD-1 inhibitors and chemotherapeutic interventions, thereby inhibiting tumor progression and metastasis. Recent investigations have also highlighted that the aging microenvironment is a significant contributor to age-related declines in immune function, offering new potential targets for restoring the immune system.

## 5. Conclusions and Future Perspectives

Immunotherapy has experienced rapid growth over the past decade, offering the advantages of relatively durable tolerability, minimal side effects, and a broad spectrum of treatments for solid tumors. Unlike conventional chemotherapy and targeted therapies, it does not depend on the cancer cells’ oncogenicity to specific dynamic pathways, such as KRAS mutations. In recent years, there has been progress in repurposing existing drugs for new indications in solid tumors; for example, in 2022, Durvalumab was approved not only for biliary tract cancer but also in combination with Tremelimumab for unresectable HCC. Additionally, therapies previously at a developmental bottleneck have gained approval for solid tumor treatments, such as the first TCR-T drug, Tebentafusp, developed in 2022 for uveal melanoma, and the first TILs drug, lifileucel, developed in 2024 for unresectable or metastatic melanoma. These advances are expected to significantly contribute to tumor immunotherapy. Despite its status as a leading therapy for cancer treatment, immunotherapy still faces challenges, including a limited therapeutic scope and efficacy, high costs, susceptibility to irAEs, and drug tolerance. As the elderly population grows, immune senescence has become a critical factor limiting its effectiveness, yet the age factor is often underrepresented in preclinical studies. Age-related changes in the TME, such as the accumulation of various immunosuppressive cells, increase tumor resistance and immune evasion. A deeper understanding of the complex interactions between aging, cellular senescence, and the TME is crucial for developing more effective cancer treatment strategies and addressing the unique challenges of age-related changes.

We provide insight into the potential mechanisms and therapeutic targets of immune senescence in malignant tumors by describing the process of immune senescence and related markers. Despite significant progress in basic and clinical studies of senescence, current immunological techniques are insufficient to fully unravel the complexity of the immune system. For instance, the applicability of the six-to-eight-week-old mouse model, primarily used in tumor research, may have limitations in human studies. Discussions continue regarding the alignment of age criteria between humans and mice. Therefore, there is a need to develop more representative living models of aging that reflect changes in immune age and time series dynamics. To address the growing public health challenges posed by malignant tumors, a comprehensive understanding of immune senescence and a detailed examination of the complexity of the human immune system still necessitate the use of advanced immunological techniques and experimental tools. Currently, targeting immunosenescent cells is emerging as a novel intervention strategy for cancer therapy, offering new therapeutic opportunities for patients, and is anticipated to lead to significant breakthroughs in cancer treatment.

## Figures and Tables

**Table 1:** Summary of currently approved drugs for ICIs.

Target	Drug	Monotherapy	Combination Therapy	Clinical Treatment	Report
PD-1	Nivolumab (Opdivo)	NSCLC, HNSC, GAC and AEG, EAC, STAD, LC, ESCA, BLCA, SCLC, HCC, SKCM, HL, RCC	Fluoropyrimidines + Platinum: ESCC; Cisplatin/Carboplatin: NSCLC; Ipilimumab: NSCLC, CRC, RCC, SKCM, HCC, MPM, cSCC; Relatlimab: SKCM	Front-line treatment: MPM, STAD, AEG, ESCA; Metastatic disease: NSCLC, HNSC, STAD, ESCA; Adjuvant: ESCA, BLCA; Neoadjuvant: NSCLC; Maintenance: NSCLC, HNSC, GAC	NMPA, FDA, EMA
	Pembrolizumab (Keytruda)	SKCM, ESCC, NSCLC, HNSC, CRC, ESCA, HCC, BC, NSCLC, CHL, PMBL, BLCA, STAD, CCA, NMIBC	Pemetrexed + Carboplatinum: NSCLC; Carboplatin + Paclitaxel: NSCLC; Paclitaxel/Nab-paclitaxel/Gemcitabine + Carboplatin: TNBC; Platinum-based + Platinum-based: AEG, HNSC, ESCA; Gemcitabine + Cisplatin: BTC; Trastuzumab + Fluorouracil/Platinum: STAD, GEJ; Axitinib: RCC; Lenvatinib: UCEC	Front-line treatment: NSCLC, HNSC, CRC, ESCA, BTC; Metastatic disease: SKCM, NSCLC, ESCC, CRC; Neoadjuvant: TNBC; Maintenance: HCC, CRC	NMPA, FDA, EMA
	Cemiplimab (Libtayo)	NSCLC, cSCC, BCC		Front-line treatment: NSCLC; Metastatic disease: cSCC; Maintenance: BCC	FDA, EMA
	Dostarlimab (Jemperli)	UCEC		Maintenance: UCEC	FDA, EMA
	Retifanlimab (Zynyz)	EC, MCC, SCAC		Metastatic disease: SCAC, MCC; Maintenance: SCAC, EC	FDA
	HLX10 (Serplulimab)	SKCM, NPC, BLCA	Platinum: ESCC; Pemetrexed + Platinum: NSCLC; Axitinib: RCC	Front-line treatment: NPC, ESCC, NSCLC; Metastatic disease: SKCM, NPC, BLCA, RCC; Adjuvant: NSCLC; Maintenance: NPC, BLCA	NMPA
	Sintilimab Injection	HL	Pemetrexed + Platinum: NSCLC; Gemcitabine + Platinum: NSCLC; Bevacizumab: HCC; Cisplatin + Paclitaxel/Cisplatin + Fluoropyrimidine containing: ESCC; Oxaliplatin + Capecitabine: G/GEJ; Bevacizumab + Pemetrexed + Cisplatin: NSCLC	Front-line treatment: NSCLC, HCC, ESCA, G/GEJ; Metastatic disease: HCC, ESCA, NSCLC; Maintenance: HL, NSCLC	NMPA
	Camrelizumab (SHR-1210)	HL, HCC, ESCC, NPC	Pemetrexed + Carboplatin: NSCLC; Cisplatin + Gemcitabine: NPC; Paclitaxel + Carboplatin: NSCLC; Paclitaxel + Cisplatin: ESCC; Apatinib: HCC	Front-line treatment: NSCLC, NPC, ESCC, HCC; Metastatic disease: HCC, ESCC, NPC; Maintenance: NPC, ESCC, HCC, HL	NMPA
	Tislelizumab	HL, BLCA, HCC, NSCLC, GAC, UCEC, HCCA, PACA, SCC	Paclitaxel/Albumin Paclitaxel + Carboplatin: NSCLC; Pemetrexed + Platinum: NSCLC; Gemcitabine + Cisplatin: NPC; Fluorouracil + Platinum: G/GEJ, ESCC;	Front-line treatment: NSCLC, G/GEJ, ESCC, HCC; Metastatic disease: BLCA, NSCLC, ESCC; Maintenance: NSCLC, HL, BLCA, HCC, ESCC	NMPA
	Penpulimab (AK105)	HL	Paclitaxel + Carboplatin: NSCLC	Front-line treatment: HL; Metastatic disease: NSCLC	NMPA
	Zimberelimab (GLS-010)	r/r cHL, CCA		Metastatic disease: r/r Chl; Maintenance: CCA	NMPA
	Serplulimab (HLX10)	CRC, GAC, UCEC, G/GEJ, HCCA, PACA, HCC	Carboplatin + Albumin paclitaxel: sqNSCLC; Carboplatin + Etoposide: ES-SCLC; Fluorouracil + Platinum: ESCC	Front-line treatment: sqNSCLC, ES-SCLC, RSCC; Metastatic disease: RSCC	NMPA
	Pucotenlimab	CRC, GAC, BC, PCa, HCC, LCA, HL		Metastatic disease: CRC, GAC, BC, PCa, HCC, LCA, HL	NMPA
PD-L1	Durvalumab (Imfinzi)	NSCLC, CA, ASTS, TNBC	Etoposide + Platinum/Carboplatin: ES-SCLC; Gemcitabine + Cisplatin: BTC; Tremelimumab: HCC	Front-line treatment: NSCLS, SCLS, BTC	NMPA, FDA, EMA
	Atezolizumab (Tecentriq)	NSCLC	Carboplatin + Etoposide: SCLC; Bevacizumab: HCC; Pemetrexed + Platinum: NSCLC	Front-line treatment: SCLC, HCC, NSCLC; Adjuvant: NSCLC	NMPA, FDA, EMA
	Avelumab (Bavencio)	MCC, BLCA	Axitinib: RCC	Front-line treatment: MCC, BLCA, RCC	FDA, EMA
	Envolizumab	CRC, GAC, UCEC, G/GEJ, HCCA, PACA, HCC		Maintenance: CRC, GAC, UCEC, G/GEJ, HCCA, PACA, HCC	NMPA
	Sugilizumab	NSCLC	Paclitaxel + Carboplatin: NSCLC; Fluorouracil + Platinum: ESCC, G/GEJ	Front-line treatment: NSCLC, ESCC, G/GEJ	NMPA
	Adebelizumab		Etoposide + Carboplatin: ES-SCLC	Front-line treatment: ES-SCLC	NMPA
	Sokazolizumab	CCA		Metastatic disease: CCA	NMPA
CTLA-4	Ipilimumab (Yervoy)	SKCM	Navulizumab: MPM, NSCLC, CRC, RCC, SKCM, HCC	Front-line treatment: MPM, NSCLC, CRC, RCC; Second-line treatment: HCC	NMPA, FDA
	Tremelimumab		Durvalumab: HCC	Front-line treatment: HCC	FDA
PD-L1/ CTLA-4 Bispecific Antibody	Cardunolizumab	R/MCC		Front-line treatment: R/MCC	NMPA
LAG-3	Relatlimab		Nivolumab: SKCM	Neoadjuvant: SKCM	FDA

Abbreviation description: AEG: adenocarcinoma of the esophagogastric junction; ASTS: adult soft-tissue sarcoma; BC: breast cancer; BCC: basal cell carcinoma; BLCA: bladder urothelial carcinoma; BTC: biliary tract cancer; CCA: cervical cancer; (C)HL: (classical) Hodgkin lymphoma; cSCC: cutaneous squamous cell carcinoma; EAC: esophageal adenocarcinoma; ESCA: esophageal carcinoma; ESCC: esophageal squamous cell carcinoma; ES-SCLC: extrapulmonary small cell lung cancer; GAC: gastric adenocarcinoma; G/GEJ: adenocarcinoma of the gastroesophageal junction; HCC: hepatocellular carcinoma; HCCA: hilarcholangiocarcinoma; HL: Hodgkin lymphoma; HNSC: head and neck squamous cell carcinoma; LC: lung cancer; MCC: Merkel cell carcinoma; MPM: malignant pleural mesothelioma; NMIBC: non-muscular invasive bladder cancer; NPC: nasopharyngeal carcinoma; NSCLC: non-small cell lung carcinoma; PACA: pancreatic cancer; PCa: prostatic carcinoma; PMBL: primary mediastinal large B-cell lymphoma; RCC: renal cell carcinoma; r/r cHL: relapsed/refractory classical Hodgkin lymphoma; SCLC: small cell lung carcinoma; SCAC: squamous cell anal cancer; SKCM: skin cutaneous melanoma; sqNSCLC: squamous non-small cell lung cancer; STAD: stomach adenocarcinoma; TNBC: triple-negative breast cancer; UCEC: endometrial cancer.

**Table 2:** Comparison of CAR-T, CAR-NK, TCR-T, and TILs therapies.

	TILs	TCR-T	CAR-T	CAR-NK
Cell Source	Tumor tissue	PBMC, iPSC	PBMC, iPSC, UCB	PBMC, iPSC, hESC, UCB, BM, cell line
Theory	Extraction of TILs from tumor samples	Transduction of TCR into T cells	Transduction of CAR into T cells	Transduction of CAR by autologous NK cells or allogeneic NK cells
Gene transfer	/	Easy	Easy	Difficult
Persistence	Low	Moderate	Moderate	Low
Infiltration capacity	High	Low	Low	Low
Toxicological	Low OTOT and CRS	CRS	CRS, OTOT, ICANS, GvHD	Low OTOT CRS, ICANS, and GvHD
Dominance	Multi-target excitation; high tumor specificity; potential anti-tumor activity	Recognition against multiple tumor antigens; high number of T cells;	High number of T cells; recognition against multiple tumor antigens	Natural anti-tumor activity; no need for MHC molecules to be involved
Drawbacks	Time-consuming and costly	Poor tumor-specific binding capacity; limited by MHC	Tumor antigen heterogeneity and tumor antigen loss	Limited efficacy of CAR transduction

Abbreviation description: BM: bone marrow; hESC: human embryonic stem cells; hPsC: human pluripotent stem cells; iPSC: induced pluripotent stem cells; PBMC: peripheral blood mononuclear cell; UCB: umbilical cord blood.

**Table 3:** Summary of currently approved drugs for ACTs.

Naturopathy	Drug	Target	Disease	Favor	Time
TILs	Lifileucel	CD19	SKCM	FDA	2024
TCR-T	Tebentafusp	Gp100	UM	FDA	2022
CAR-T	Kymriah	CD19	ALL, NHL (DLBCL, HGBL)	FDA	2017
	Yescarta	CD19	NHL (DLBCL, FL, HGBL, PMBL	FDA	2017
	Tecartus	CD19	RR/MM, ALL	FDA	2020
	Breyanzi	CD19	NHL, (DLBCL, G3BFL, PMBL)	FDA	2021
	Abecma	BCMA	RR/MM	FDA	2021
	Carvykti	BCMA	RR/MM	FDA, NMPA	2022
	Axicabtagene ciloleucel	CD19	r/r LBCL (DLBCL, PMBL, HGBL, DLBCL)	NMPA	2021
	Carteyva	CD19	r/r LBCL	NMPA	2021
	Equecabtagene autoleucel	BCMA	RR/MM	NMPA	2023
	Inaticabtagene autoleucel injection	CD19	r/r B-ALL	NMPA	2023
	CT053 (Zevorcabtagene autoleucel)	BCMA	RR/MM	NMPA	2023

Abbreviation description: ALL: acute lymphoblastic leukemia; BCMA: B cell maturation antigen; CD19: cluster of differentiation 19; DLBCL: diffuse large B-cell lymphoma; FL: follicular lymphoma; G3BFL: diffuse large B-cell lymphoma, grade 3, with Burkitt-like features; Gp100: glycoprotein 100; HGBL: high-grade B-cell lymphoma; NHL: non-Hodgkin’s lymphoma; PMBL: primary central nervous system lymphoma; r/r B-ALL: relapsed/refractory B-cell acute lymphoblastic leukemia; r/r LBCL: relapsed/refractory large B-cell lymphoma; RR/MM: relapsed/refractory multiple myeloma; UM: uveal melanoma.

**Table 4:** Registered ongoing clinical trials of TILs, CAR-T, CAR-NK, and TCR-T therapies for solid tumors on Clinicaltrials.gov.

NCT– Number	Study Start	Tumor Type	Interventions	Phase	Country
TILs					
NCT05475847	2022.07	CCA	ATIL (C-TIL052A) Injection	1	China
NCT05878028	2022.09	NSCLC	L-TIL + Tislelizumab + Docetaxel	2	China
NCT05451784	2022.07	TNBC-METS	PD1+ TILs (NUMARZU-001)	2	Spain
NCT05676749	2024.02	MNSCLC	C-TIL051	1	USA
NCT05438797	2021.04	APCAN	Adoptive TIL-TCM transfer therapy	1	
NCT05681780	2023.01	NSCLC	TIL, Nivolumab, Cyclophosphamide	2	USA
NCT05869539	2023.06	AM	TIL t+ ANV419	1	Switzerland
TCR-T					
NCT05438667	2022.06	PACA	TCR-T therapy	1	China
NCT06119256	2023.08	EBV-AIAHSCT	EBV-TCR-T cells	1	China
NCT06135922	2023.08	EBV-HLH	EBV-TCR-T cells	1	China
NCT04509726	2023.03	NPC	EBV-specific TCR-T cell	2	China
NCT05122221	2022.07	CCA, HNC, ANAL-CA	Fludarabine + Cyclophosphamide, Interleukin-2, CRTE7A2-01 TCR-T Cell	1	China
NCT04520711	2022.02	MEN	TCR-transduced T cells, CDX-1140, Pembrolizumab	1	USA
CAR-T					
NCT06132711	2023.11	MM	APRIL-BAFF-Bicephali CAR-T cells	2	China
NCT05420493	2021.09	RNHL, RNFHL, NHL	CAR-T cells	1	China
NCT05749133	2023.04	MM	CAR-T Cells Injection	2	China
NCT06429150	2024.05	MM, PCT	CAR-T cells	2	Russian
NCT05596266	2022.10	T-ALL	CD5 CAR-T	1	China
NCT05333302	2020.10	B-ALL, LBCL	CD19 CAR-T-cells, Tocilizumab	1	Belarus
NCT05535855	2024.01	ALL	CD19 Directed CAR T Cell	1	USA
CAR-NK					
NCT05472558	2022.09	BNHL	Anti-CD19 CAR-NK	1	China
NCT05673447	2023.03	DLBCL	Anti-CD19 CAR NK cells	1	China
NCT04847466	2021.12	GEJ, HNSCC	N-803, Pembrolizumab	2	USA
NCT06325748	2024.07	AML, MDS	SENTI-202	1	USA
NCT06045091	2023.06	MM, PCL	CAR-NK cells injection	1	China
NCT06421701	2024.06	SLE	Anti-CD19 CAR-NK cells	1	China
NCT06307054	2024.03	AML	Anti-CLL-1 CAR NK cells	1	China

Abbreviation description: AM: advanced melanoma; AML: acute myeloid leukemia; ANAL-CA: anal cancer; APCAN: advanced pancreatic cancer; ATIL: autologous tumor infiltrating lymphocytes; BNHL: B-cell non-Hodgkin lymphoma; EBV-AIAHSCT: Epstein–Barr virus infection after allogenic hematopoietic stem cell transplantation; HLH: hemophagocytic lympho-histiocytosis; HNC: head and neck cancers; HNSCC: head and neck squamous cell carcinoma; MDS: myelodysplastic syndromes; MEN: malignant Epithelial Neoplasms; MNSCLC: metastatic non-small cell lung cancer; PCL: plasma cell leukemia; PCT: plasmacytoma; RNFHL: refractory non-Hodgkin lymphoma; RNHL: relapsed non-Hodgkin lymphoma; SLE: systemic lupus erythematosus; T-ALL: T-cell acute lymphoblastic leukemia; TNBC-METS: metastatic triple-negative breast carcinoma.

**Table 5:** A key indicator of immunosenescence is thymic degeneration, associated with reduced IL-7 secretion. As the immune system remodels with age, notable changes include the loss of CD27 and CD28 expression and an increase in CD57 and KLRG-1 expression. Aging also enhances glycolysis and ROS production while decreasing mitochondrial synthesis, alongside reduced telomere length and telomerase activity. Consequently, clinical trials and therapies targeting these markers of immune senescence have commenced.

Treatment	Clinical Trial/Drugs	Target	References
IL-7	IL-7	Thymic	[162]
KGF (keratinocyte growth factor)	KGF	Thymic	[162]
IL-22	IL-22	Thymic	[162]
Ghrelin	Ghrelin	Thymic	[162]
Third-gen CAR-T cells containing CD28 + CD137	NCT02186860	CD28	[163]
Second-gen CMV-selected CAR-T cells against HER2 containing CD28.zeta signaling domain	NCT01109095	CD28	[163]
TAB08	NCT01990157	CD28	[163]
Rapamycin	Rapamycin	MTOR	[157]
Metformin	Metformin	Mitochondrial respiration	[157]

## Data Availability

Not applicable.

## Abbreviations

CD66a	Carcino-Embryonic Antigen-related Cellular Adhesion Molecule1, CEACAM1
Galectin-9	Lectin, Galactoside-Binding, Soluble 9
HMGB1	High-Mobility Group Box 1
PS	Phosphatidylserine
